# California Letter

**Published:** 1882-04

**Authors:** 


### Domestic Correspondence.

### ARTICLE VI.

LOS ANGELOS, CAL., March 1st, 1882.

The climate of Southern California is delightful; everybody who comes here is pleased with it. This alone is sufficient to account for much of the benefit which invalids here experience—and the invalids who come here for health nearly all improve, except those who are in an advanced stage of pulmonary consumption when they arrive. The climate is semi-tropical; there is no winter—as we in Chicago understand it—and there is no tropical summer; the summers are no hotter than they are on Lake Michigan. The year 1880 was an exceptional one, with numbers of severe frosts and of fogs. I inclose the meteorological report of that year for this place—made by the Government observer.

The frosts referred to were simple hoar-frost, which killed the flowers and tender leaves. This winter there have been several such frosts, but all so slight that the calla-lilies, the roses and heliotropes do not show perceptible damage. The fogs generally occur in the early morning and are gone soon after sunrise. Riding, early in February, over a plain near here, where the grass was just springing up and the air was balmy and warm, one of our party instinctively remarked: "What a cold and desolate spot this must be in Winter!"

With so slight a rain-fall there is a dearth of vegetation on the hills and valleys except where irrigation is practiced. The unirrigated land is used for sheep grazing mostly. In the very dry seasons many sheep are lost by starvation—we have seen some dozens of such in our drives hereabout. Water the soil and it will produce almost anything. The valleys are being rapidly

### LOS ANGELOS METEOROLOGICAL SUMMARY 1880—MONTHLY AND ANNUAL MEANS.

| DATE. | Barometer | Highest Barometer | Lowest Barometer | Range Barometer | Mean Therm. | Highest Therm. | Lowest Therm. | Range of Temp. | Relative Humidity per cent. | Prevailing Wind Direction. | Total No. Miles. | Maximum Wind Velocity. | Amount Rainfall | No. Days Rainfall |
|---|---|---|---|---|---|---|---|---|---|---|---|---|---|---|
| January | 30.065 | 30.304 | 29.692 | 0.612 | 50.5 | 76.0 | 30.0 | 46.0 | 65.0 | N. | 3214 | 21 | 1.33 | 5 |
| February | 30.149 | 30.387 | 29.876 | 0.511 | 49.1 | 70.5 | 33.5 | 37.0 | 67.0 | NE. | 3700 | 26 | 1.56 | 8 |
| March | 30.068 | 30.312 | 29.872 | 0.440 | 50.5 | 73.5 | 36.0 | 37.5 | 72.6 | NE. | 3622 | 17 | 1.45 | 6 |
| April | 30.076 | 30.219 | 29.936 | 0.283 | 55.2 | 83.0 | 40.0 | 43.0 | 77.4 | SW. | 4073 | 23 | 5.06 | 13 |
| May | 29.951 | 30.160 | 29.782 | 0.378 | 61.3 | 97.0 | 42.0 | 55.0 | 72.0 | SW. | 3508 | 19 | .04 | 1 |
| June | 29.914 | 30.014 | 29.800 | 0.214 | 62.9 | 83.0 | 50.0 | 33.0 | 72.4 | SW. | 3185 | 14 | .00 | 0 |
| July | 29.961 | 30.071 | 29.859 | 0.212 | 63.5 | 85.0 | 52.0 | 33.0 | 73.4 | SW. | 3377 | 22 | .00 | 0 |
| August | 29.871 | 30.072 | 29.681 | 0.391 | 66.1 | 87.0 | 52.0 | 35.0 | 71.9 | SW. | 3178 | 19 | .00 | 0 |
| September | 29.929 | 30.059 | 29.794 | 0.265 | 63.7 | 91.0 | 44.0 | 47.0 | 71.3 | SW. | 2694 | 14 | .00 | 0 |
| October | 29.997 | 30.152 | 29.850 | 0.302 | 61.6 | 89.0 | 44.0 | 45.0 | 62.5 | SW. | 2423 | 16 | .14 | 2 |
| November | 30.067 | 30.314 | 29.756 | 0.558 | 54.8 | 85.0 | 35.0 | 50.0 | 55.1 | NE. | 1918 | 17 | .67 | 3 |
| December | 30.040 | 30.269 | 29.647 | 0.622 | 55.0 | 80.0 | 38.0 | 42.0 | 71.6 | NE. | 2403 | 22 | 8.40 | 13 |
| Sums | 360.088 | 362.333 | 357.545 | 4.788 | 704.2 | 1000.0 | 496.5 | 503.5 | 832.2 | | 37,295 | 230 | 18.65 | 51 |
| Means | 30 007 | 30.195 | 29.795 | 0.400 | 58.7 | 83.3 | 41.4 | 41.9 | 69.3 | SW. | | 19.2 | | |

### GENERAL REMARKS.

January—Damaging frosts end of month, range of temperature above normal.

February—Frosts, not damaging. Maximum temperature, 9.5 ; lower than normal.

March—Frosts 5th, 15th and 16th. No damage. Weather unusually disagreeable for corresponding month.

April—Frost, 19th, no injury. Maximum temperature on 9th above normal.

May—Frequent fogs during month ; temperature above normal.

June—Weather hazy and foggy; temperature below normal.

July—Dense wet fogs; maximum thermometer, 23d, and minimum thermometer, 22d of month.

August—Season backward; maximum thermometer, 5th; minimum thermometer, 6th.

September—Frequent fogs; maximum thermometer, 5th; minimum thermometer 23d and 29th.

October—Weather warm and pleasant; maximum thermometer, 16th; minimum thermometer, 11th.

November—First frost, 13th; no damage; maximum thermometer, 4th; minimum thermometer, 13th and 16th.

December—Frost, 1st; rains of great benefit; maximum thermometer, 11th; minmum thermometer, 1st.

improved, and irrigating ditches or zanzas—pronounced *sanka*—are being extended in every direction. It is very dusty here in the summer of course, but we have seen very little of it. The streets of this city are sprinkled the year round. The scenery is beautiful; the grandest sight is the snow-covered Sierras viewed over the tropical vegetation—in the foreground—of flowers, orange trees loaded with yellow fruit so heavily that the limbs must often be supported to prevent their breaking; magnolia, lemon, lime, cyprus, pepper and eucalyptus trees, all clothed in in the richest of green leaves. The population of the towns in Southern California, aside from the original Mexican inhabitants of the older towns, is made up largely of people who have migrated here in search of health. Those who have improved, which are a majority, are so fulsome in their laudation of the climate and its benefits to the sick that you need to allow liberally for their enthusiasm.

This climate is a good one for invalids—probably not for all classes of invalids, and certainly not for those far advanced in phthisis. But for incipient phthisis, bronchitis, general debility, neurasthenia, neuralgia, dyspepsia and catarrh, it must be that a change from a northern climate to this is very beneficial. It is impossible that so much and so uniform testimony from so many people who have proved it experimentally can be unreliable.

Nor does one care much, in the face of such testimony, for theories about the climate based on a study of the meteorological tables unless the theories are favorable; one feels like saying, if the theories make out the climate a bad one, "so much the worse for the theories."

But in coming here for health one should exercise some wisdom in several directions. If he is very ill he should not come alone; some friend should come with him to find a boarding place and take care of him for a time at least. Then, he should be sure that, wherever he goes to live, or stay for one day even, he has a fire in his room whenever he feels cold. The air becomes chilly late every afternoon, especially in winter, and a Northern man needs his overcoat if out of doors, and a fire if indoors, as night approaches. The old residents have become so used to the climate that they don't think of having a fire unless the temperature falls to near freezing point. They will build a house of a dozen rooms and provide for a fire in just two—in all the rest you cannot find a fireplace, a stovepipe hole or even a chimney. Phthisical patients who have passed beyond the early stage of the disease should not come here to stay. This is the largest city in Southern California, and offers perhaps better advantages for the entertainment of health seekers than any other, but there are eight or ten other towns in this quarter where one may make himself comfortable if he only has money. The best way to do is to make this headquarters and visit other regions as one's inclination prompts. The best way to reach here, except in the summer months, is by the new Southern Pacific and Santa Fe route; in summer the Union and Central Pacific Railways are the best.

The mortality in Los Angelos is relatively high—for the month of February ult. it represented an annual death rate of 26 per 1000. The proportion of deaths from phthisis is large and swells the list materially, but most of the cases are in people who have been here less than a year, thus testifying to the fact that many come here with that disease to die. Malarial fevers occur and add to the mortality list, also typhoid fever, which has been rather prevalent of late.

New Mexico and Arizona offer doubtless a more ideal climate for consumptives, especially in the advanced stages, than this region. The trouble is that in the territories referred to there are few places where invalids enjoy living which offer any real attractions for that class of sick people who are able to travel for health. Los Vegas, N. M., has some hot springs that are highly recommended, but very few attractions beside.

We spent a day at Santa Fe, and were very much entertained with the mud houses, the donkeys and the old churches, something less than six centuries old; also with the pleasant scenery in the distance, but one can *do* the town in a few hours, and it is no wonder invalids tire of the place for want of pleasant surroundings and diversion. The time will come probably when many more attractions will call visitors to this old town and beguile them to remain more than a brief day.

Sick people who are sent away from home to get well, or drag out a longer existence in their invalidism than they could at home, must go where they can find some enjoyment if they are to receive the greatest benefit, or much benefit. No health resort will attain much popularity till it becomes enjoyable. Here is one that is in a very high degree enjoyable.

In nearly every public hospital in California—if not every one—and in many private hospitals, the physicians and surgeons receive pay for their services. In the Hospital of the " City and County of San Francisco " each attending physician and surgeon receives $1,200 annually for his services. In the German Hospital each one receives $900. The resident physician at the County Hospital receives $1,500, while at the German Hospital this officer draws but $1,200, but in each case he is debarred from engaging in any private practice. In the French Hospital, with 160 beds, there is one attending physician, who is on duty throughout the year. He is supposed to visit the institution at least once each day, and his pay is $1500.

In nearly or quite all the county hospitals throughout the State a salary attends the office of physician. The amount varies, outside of San Francisco, from $300 to $1,000, the latter figure being the pay in the county of Los Angelos. Some of the county hospitals are at the same time almshouses, the county caring for its poor, sick and well, under the same roof; but in some cases the attending physician receives additional compensation as superintendent of the poor-house part of the establishment.

The " County and City Hospital " is under the management of the Board of Health of San Francisco, appointed by the Governor of the State. They appoint all the officers, medical officers included. The Board of Supervisors hold the purse and vote the appropriations, but have no further voice in the management. The attending physicians and surgeons are chosen from the faculties of the two medical colleges—the colleges being equally represented; the representatives of each college have exclusive control of an equal number of wards, and two internes are chosen annually from the graduates of each college, to serve one year. A superintendent physician, who lives and practices outside, and who gives only an hour or two to the hospital daily, has charge of the institution; the house physician lives in the building and gives his whole time to his duties.

There is a small amphitheater for clinics and here the students of the two colleges receive their hospital instruction. One college, the younger, the "Medical College of the Pacific," is three miles away from the Hospital; the older, the "Medical Department" of the State University (the old Toland School), is nearly four miles away.

The County Hospital is well managed. It is kept clean and in fair repair by the labors of the patients alone. No histories or notes of cases are kept on tablets hung over the beds, but only the name, age, residence, diagnosis, etc., of each patient. The histories are kept in books for the purpose.

Liquor is issued to patients only on the written order of the house physician and the order must be approved in writing by the attending physician within twenty-four hours.

There are several private hospitals in San Francisco I was unable to visit. I regretted this very much, and in particular that time did not permit a visit to one on Falsom street which supports a sign: "*Private Female Lying-In Hospital.*" There is nothing, as Dr. J. S. Billings would say, like giving an *article* a proper title. N. B.

